# Role of *sph2* Gene Regulation in Hemolytic and Sphingomyelinase Activities Produced by *Leptospira interrogans*

**DOI:** 10.1371/journal.pntd.0003952

**Published:** 2015-08-14

**Authors:** Suneel A. Narayanavari, Kristel Lourdault, Manjula Sritharan, David A. Haake, James Matsunaga

**Affiliations:** 1 Department of Animal Biology, University of Hyderabad, Hyderabad, India; 2 Research Service, Veterans Affairs Greater Los Angeles Healthcare System, Los Angeles, California, United States of America; 3 Department of Medicine, David Geffen School of Medicine at University of California, Los Angeles (UCLA), Los Angeles, California, United States of America; 4 Department of Urology, David Geffen School of Medicine at University of California, Los Angeles (UCLA), Los Angeles, California, United States of America; 5 Department of Microbiology, Immunology and Molecular Genetics, University of California, Los Angeles, Los Angeles, California, United States of America; 6 Division of Infectious Diseases, Veterans Affairs Greater Los Angeles Healthcare System, Los Angeles, California, United States of America; Institut Pasteur, FRANCE

## Abstract

Pathogenic members of the genus *Leptospira* are the causative agents of leptospirosis, a neglected disease of public and veterinary health concern. Leptospirosis is a systemic disease that in its severest forms leads to renal insufficiency, hepatic dysfunction, and pulmonary failure. Many strains of *Leptospira* produce hemolytic and sphingomyelinase activities, and a number of candidate leptospiral hemolysins have been identified based on sequence similarity to well-characterized bacterial hemolysins. Five of the putative hemolysins are sphingomyelinase paralogs. Although recombinant forms of the sphingomyelinase Sph2 and other hemolysins lyse erythrocytes, none have been demonstrated to contribute to the hemolytic activity secreted by leptospiral cells. In this study, we examined the regulation of *sph2* and its relationship to hemolytic and sphingomyelinase activities produced by several *L*. *interrogans* strains cultivated under the osmotic conditions found in the mammalian host. The *sph2* gene was poorly expressed when the Fiocruz L1-130 (serovar Copenhageni), 56601 (sv. Lai), and L495 (sv. Manilae) strains were cultivated in the standard culture medium EMJH. Raising EMJH osmolarity to physiological levels with sodium chloride enhanced Sph2 production in all three strains. In addition, the Pomona subtype kennewicki strain LC82-25 produced substantially greater amounts of Sph2 during standard EMJH growth than the other strains, and *sph2* expression increased further by addition of salt. When 10% rat serum was present in EMJH along with the sodium chloride supplement, Sph2 production increased further in all strains. Osmotic regulation and differences in basal Sph2 production in the Manilae L495 and Pomona strains correlated with the levels of secreted hemolysin and sphingomyelinase activities. Finally, a transposon insertion in *sph2* dramatically reduced hemolytic and sphingomyelinase activities during incubation of *L*. *interrogans* at physiologic osmolarity. Complementation of the mutation with the *sph2* gene partially restored production of hemolytic and sphingomyelinase activities. These results indicate that the *sph2* gene product contributes to the hemolytic and sphingomyelinase activities secreted by *L*. *interrogans* and most likely dominates those functions under the culture condition tested.

## Author Summary

The spirochete *Leptospira* causes leptospirosis, a potentially deadly disease of humans and animals. Candidate factors that promote infection include hemolysins encoded by several leptospiral genes. Hemolysins rupture red blood cells *in vitro*. Some hemolysins are sphingomyelinases, which target sphingomyelin in the host cell membrane. Hemolysins have the potential to disrupt organ function during infection. It is not known which hemolysins and sphingomyelinases are responsible for the hemolytic and sphingomyelinase activities secreted by *L*. *interrogans*. We found that the production of hemolytic activity is regulated and is tied to expression of *sph2*, which encodes a hemolysin with sphingomyelinase, cytotoxic, and fibronectin-binding activities. Hemolytic and sphingomyelinase activities and *sph2* expression were higher when the osmolarity of the culture medium was raised to the level found in the mammalian host. Similarly, *sph2* expression was substantially higher in an *L*. *interrogans* strain that secreted large amounts of hemolytic and sphingomyelinase activities than in a strain that generated negligible amounts. Most importantly, disruption of the *sph2* gene eliminated hemolysin production and yielded substantially less sphingomyelinase than the wild-type strain. Our findings indicate that *sph2* is a major contributor to the hemolytic and sphingomyelinase activities secreted by *L*. *interrogans* and that the hemolytic and sphingomyelinase activities measured in standard *L*. *interrogans* cultures may underestimate the levels produced during infection.

## Introduction

Leptospirosis is a neglected zoonotic disease that afflicts humans and animals [1–3]. Although the disease occurs worldwide, it is observed most frequently in tropical countries, where conditions for environmental survival and transmission are most favorable [4]. The causative organisms are spirochetes belonging to the genus *Leptospira*, which comprise pathogenic and nonpathogenic species. The bacteria are able to enter the mammalian host through skin abrasions and mucous membranes. Following entry, the spirochetes disseminate via the bloodstream to many organs [2]. Patients exhibit a wide range of signs and symptoms from undifferentiated fever to liver dysfunction, renal insufficiency, hemolytic anemia, bleeding, and respiratory failure [5]. Proposed mechanisms of pathogenesis include vascular damage, which is frequently manifested as hemorrhage in the lungs and other organs on post-mortem examination [6]. Additionally, reproductive failure is observed in livestock animals with leptospirosis [7]. *Leptospira* bacteria are spread and maintained in the environment by rats and other reservoir hosts, whose renal tubules are persistently colonized by the spirochete. Humans and animals can be infected by direct or indirect contact with contaminated urine or other tissue.

Many *Leptospira* strains secrete hemolysins and sphingomyelinases during *in vitro* growth [8–11]. Strains of serovar Pomona secrete the highest levels of hemolytic activity [9] and cause hemolytic anemia in ruminants [12–14]. The single peak of hemolytic activity detected by isoelectric focusing of the culture supernatant fluid of one Pomona strain co-purified with sphingomyelinase C activity, suggesting that hemolysis is due to sphingomyelinase [15]. It is not known which gene or genes encode the secreted hemolytic and sphingomyelinase activities.

Hemolytic activity has been reported for purified recombinant forms of the *L*. *interrogans* protein HlyX, HlpA, TlyA, and the sphingomyelinase-like paralogs Sph1, Sph2, Sph3, and SphH [16–19]. Among the sphingomyelinase paralogs, sphingomyelinase C activity has been demonstrated only for Sph2, which cleaves sphingomyelin to ceramide and phosphocholine [20]. Sphingomyelinase activity has also been reported for Sph1, Sph3, and Sph4 [21], although these proteins lack some of the catalytic amino acid residues required for enzymatic activity [22]. The genes encoding the sphingomyelinase-like proteins are missing from the nonpathogen *Leptospira biflexa*. Similarly, sphingomyelinase activity has been detected only in pathogenic strains of *Leptospira* [10]. These observations suggest that sphingomyelinase-like proteins function during infection [23].

Hemolysins are assumed to assist microbial pathogens in acquiring iron by lysing host erythrocytes during infection [24]. Heme can be used by *L*. *interrogans* as an iron source for growth [25]. They express the hemin-binding protein HbpA, which may participate in transporting heme into the cell, and a heme oxygenase, which is required for heme utilization and for virulence in the hamster model of leptospirosis [26–28]. Iron depletion resulted in increased levels of a sphingomyelinase-like protein, which is consistent with the notion that hemolysins are involved in iron acquisition by *L*. *interrogans* [29]. Nevertheless, experimental evidence indicates that leptospiral hemolysins are capable of pathogenic processes that do not involve red blood cells. For example, recombinant Sph2 protein is cytotoxic towards equine endothelial cells, mouse lymphocytes and macrophages, and a human liver cell line [18, 30]. Additionally, *L*. *biflexa* ectopically expressing *sph2* captures plasma fibronectin *in vitro* [31].

Sph2 has not been detected in appreciable amounts in cell lysates of *L*. *interrogans* grown in the standard culture medium EMJH except in strains of serovar Pomona [32]. However, *sph2* expression in the Fiocruz L1-130 strain is dramatically upregulated by environmental conditions that occur during infection. In our previous whole-genome microarray study examining the changes in transcript levels to an increase in osmolarity from low osmolarity found in EMJH medium to physiologic levels found in the mammalian host, *sph2* was the second most strongly upregulated gene in the Fiocruz L1-130 strain [33]. Levels of cellular and extracellular Sph2 in the Fiocruz L1-130 strain were also upregulated upon addition of sodium chloride or sucrose to physiological levels of osmolarity [34]. Anti-Sph2 antibody is detected in patients with leptospirosis, suggesting that *sph2* is expressed during infection [32]. In a recent RNA-seq experiment, *sph2* transcript levels were higher in *L*. *interrogans* bacteria growing in dialysis membrane chambers in rats than in those growing in *in vitro* culture [35]. These observations suggest that upregulation of *sph2* is involved in *L*. *interrogans* infection.

The enzymatic domain of Sph2 is flanked by unusual sequences missing from bacterial sphingomyelinases whose crystal structures have been determined [20]. Three or four ~20 residue imperfect tandem repeats are present to the amino terminus of Sph2, and the carboxy terminus comprises a unique segment of ~186 amino acid residues [18, 22]. The enzymatic domain may not be sufficient for hemolytic activity of Sph2. A sphingomyelinase with a similar C-terminal extension is found in the sphingomyelinase protein of a fish isolate of *Pseudomonas* species [36]. Removal of the C-terminal domain eliminates its hemolytic activity despite its enzymatic domain being left intact [36]. *L*. *interrogans* releases Sph2 in smaller forms whose apparent molecular masses are ~13 and ~21 kDa less than that of the cell-associated form of Sph2 [34]. These observations raise the question of whether *L*. *interrogans* secretes Sph2 in an active form because the hemolytic activity of Sph2 was demonstrated with full-length recombinant protein instead of the shortened forms detected in culture supernatant fluids [16, 18, 34]. One approach to establish that Sph2 contributes to the secreted hemolytic activity is to demonstrate inhibition of hemolysis when Sph2 antibody is added to the spent culture medium. However, both anti-Sph2 antiserum and normal serum inhibited the hemolytic activity secreted by a Pomona strain of *L*. *interrogans* [32].

In this study, we extend our earlier findings of osmotic induction of *sph2* expression with the Fiocruz L1-130 strain to show that the *sph2* genes of four additional strains of *L*. *interrogans* are also regulated by sodium chloride, including a Pomona strain that displays high levels of basal *sph2* expression. In addition, we demonstrate that the levels of secreted hemolytic and sphingomyelinase activities are regulated by osmolarity. In our attempts to maximize *sph2* expression, we also found that rat serum increased Sph2 production further at physiologic osmolarity, although the presence of serum inhibited hemolytic activity in liquid-phase assays. Finally, we present genetic evidence that *sph2* contributes to the hemolytic and sphingomyelinase activities secreted from *L*. *interrogans*.

## Materials and Methods

### Bacterial strains and culture conditions

The leptospiral strains *Leptospira interrogans* serovars Manilae (strain L495), Pomona subtype kennewicki (strain LC82-25), and Lai (strains 56601 and L391) were grown at 30°C in EMJH medium assembled with Probumin BSA (Vaccine Grade, lot 103) as described previously [37] or purchased as Probumin Vaccine Grade Solution (lot 103) from EMD Millipore (Temecula, California, USA). The Pomona LC82-25 and Lai 56601 strains were obtained from Rich Zuerner (National Animal Disease Center, Ames, Iowa, USA) and Mathieu Picardeau (Institut Pasteur, Paris, France), respectively. *Leptospira interrogans* serovar Copenhageni strain Fiocruz L1-130 was grown at 30°C in EMJH medium supplemented with 1% heat-inactivated rabbit serum (Rockland; Gilbertsville, Pennsylvania, USA). The L391 strain and an L391 mutant with a *kan* ^*r*^-marked *Himar1* transposon inserted in the *sph2* gene were provided by Gerald Murray [38]. The *sph2* mutant was maintained in EMJH containing 40 μg/mL kanamycin. Culture densities were determined by directly counting the number of *Leptospira* using an AxioLab A1 microscope with a darkfield condenser (Zeiss Microscopy Division; Pleasanton, California, USA). To examine the influence of environmental conditions on *sph2* expression, *L*. *interrogans* cells were grown to a cell density of 0.6–1 x 10^8^ cells/mL and then supplemented with 120 mM NaCl, 10% rat serum (Rockland Immunochemicals, Limerick, Pennsylvania, USA), or a mixture of nine parts of EMJH supplemented with 120 mM NaCl and one part of rat serum and allowed to incubate for 4 h. Cultures were harvested by centrifugation at 9,200 x g for 20 min at 4°C in a Sorvall high-speed centrifuge (Thermo Scientific; Marietta, Ohio, USA), and the spent medium was collected for storage. Cells were washed once with cold PBS-5 mM MgCl_2_. Sodium chloride was added to the spent medium obtained from the EMJH cultures to equalize the osmolarity across all samples. Cell pellets and spent growth medium were stored in -80°C.

### Immunoblot analysis

For Western blots, the proteins were fractionated on 10% PAGEr Gold precast Tris-Glycine gels (Lonza; USA). Dual Color Precision Plus Protein Standards from Bio-Rad (catalog # 161–0374) (Hercules, California, USA) were included in adjacent lanes. Proteins were transferred from gels on to PVDF Immobilon-P transfer membrane (EMD Millipore; USA) using Trans-blot SD Semi-Dry transfer Cell system (Bio-Rad) (10V for 45 min). The membranes were incubated in blocking solution (5% skim milk in PBS-0.05% Tween 20 [PBS-T]) for 30 min and then incubated with rabbit anti-Sph2 and anti-LipL41 antisera [34, 39] at dilutions of 1:1,000 and 1:10,000, respectively, for 30 min and washed three times (5 min each) with PBS-T. The membrane was then incubated with donkey anti-rabbit antibody (1:5,000) (Amersham Biosciences; Piscataway, New Jersey, USA) or protein A-horseradish peroxidase conjugate (1:3,000) (Amersham Biosciences) in blocking solution for 30 min and again washed three times with PBS-T. The membranes were developed with the ECL Western blot detection system (Thermo Scientific), and the bands were visualized with Hyperfilm (Amersham Biosciences).

Immunoprecipitation of Sph2 from the spent culture medium was performed as described [37], except the culture supernatant fluid was preadsorbed with 25 μL of EZview Red Protein A Affinity gel (Sigma-Aldrich; St. Louis, Missouri, USA) for 60 min at 4°C prior to immunoprecipitation with anti-Sph2 antiserum [32]. The resuspension volumes of the immunoprecipitates with Laemmli sample buffer were adjusted according to the cell densities of the cultures.

### Plasmid DNA

Restriction enzymes and T4 DNA ligase were obtained from New England Biolabs (Beverly, Massachusetts, USA). PCR amplifications were conducted with Phusion DNA polymerase (Thermo Scientific). *L*. *interrogans* sequences cloned into plasmid vectors were verified by Sanger sequencing (Laragen; Culver City, California, USA).

The *sph1*, *sph3*, and *sph4* sequences were amplified by PCR with the primer pairs lic12632(Nd)-3F and lic12632(Xh)-4R, lic13198(Nd)-3F and lic13198(Xh)-4R, and lic11040(Nd)-3F and lic11040(Xh)-4R, respectively (Table 1). Forward and reverse primers included *Nde*I and *Xho*I restriction sites near the 5' ends. Genomic DNA from *L*. *interrogans* Fiocruz L1-130 served as template. Each amplicon was digested with *Nde*I and *Xho*I and ligated to pET20b+ (EMD Biosciences; La Jolla, California USA) that had been digested with the same enzymes. The *sph1*, *sph3*, and *sph4* expression plasmids were named pRAT547, pRAT548, and pRAT549, respectively. The *sph2* expression plasmid pTOPO-Sph2(27–623) was described previously [34].

**Table 1:** List of oligonucleotides for PCR.

Gene	Primer name*	Sequence (5’to 3’)^#^	Role
*sph1*	lic12632(Nd)-3F	CTTACAcatatgAAACCTGGCAAACAAAATTCCATAAATC	P
	lic12632(Xh)-4R	GCActcgagATGATGATAGATTAAATCTTTACTCCA	
*sph2*	lic12631(Kp)-17F	GACCATggtaccAGCGAGACGCTGAGTCTGA	P
	lic12631(Xh)-18R	GTCCATctcgagGGTATTTTATTGAATAAGATTAGGAAAGT	
*sph3*	lic13198(Nd)-3F	AATTGTcatatgAAAGATTCATTAGTATACAATAAATTTTTA	P
	lic13198(Xh)-4R	GCActcgagACGATAAATTAGATCCTTGCTCCAATCT	
*sph4*	lic11040(Nd)-3F	CTATCTcatatgGATTGTTTACCTGATTCACAGTCTTCTG	P
	lic11040(Xh)-4R	CCTctcgagTTCCTCAGGGCCTTCATTCAATTGAACA	
*sph1*	lic12632-7F	TGAAACCGTTTTGATTACGGGTGA	Q
	lic12632-8R	CGGCAAGTGCGTTTGTTTTTGTAT	
*sph2*	lic12631-11F	ATGTTGCCCACAAATCTTCCACG	Q
	lic12631-12R	CCACATCTGTTTGATACGGATATTCTTCT	
*lipL41*	lipL41-7F	TCGGAAATCTGATTGGAGCGGAAGCA	Q
	lipL41-8R	AGAAGCGGCGAAACCTGCCACT	

* F, forward primer; R, reverse primer

^#^ Restriction sites in lower case

P, plasmid construction; Q, quantitative RT-PCR

To construct the *sph2* complementation plasmid, the oligonucleotides lic12631(Kp)-17F and lic12631(Xh)-18R (Table 1) were used to PCR amplify *sph2* along with its flanking regions using Fiocruz L1-130 genomic DNA as template. The amplicon included sequences 327 bp upstream and 130 bp downstream of the start and stop codons, respectively. Restriction sites for *Kpn*I and *Xho*I were included near the 5' ends of the oligonucleotides. Following digestion of the amplicon with *Kpn*I and *Xho*I, the *sph2*-containing fragment was inserted into the similarly-digested plasmid pRAT575 [40] to create pRAT613 for the current and future studies. The *Kpn*I-*Xho*I fragment containing *sph2* was subsequently transferred to the mobilizable plasmid pAL614, which contains *Kpn*I and *Xho*I sites between the ends of a *Himar1* element that also harbored a gene encoding resistance to spectinomycin [41]. The resulting *sph2* plasmid was designated pRAT708.

### Recombinant protein expression and purification

Frozen competent *E*. *coli* BLR(DE3)/pLysS (EMD Biosciences) was transformed with the plasmids pRAT547 (*sph1*), pRAT548 (*sph3*), pRAT549 (*sph4*), and pTOPO-Sph2(27–623) (*sph2*), and transformants were selected on LB plates containing 100 μg/mL carbenicillin. Colonies were inoculated into 10 mL LB containing carbenicillin for overnight growth at 37°C. 5 mL of each overnight culture was then placed into 200 mL LB with carbenicillin and incubated at 37°C. When the OD at 600 nm reached 0.6, 1 M IPTG was added to a final concentration of 0.5 mM. For expression of recombinant Sph1, Sph3, and Sph4, the cultures were incubated for 2 hrs at 37°C. The bacteria were collected by centrifugation, and the cell pellets were stored at -80°C. For expression of recombinant Sph2, the culture was incubated overnight at 16°C and harvested by centrifugation. To lyse the bacteria, cell pellets were suspended in 5 mL (for Sph1, Sph3, and Sph4) or 10 mL (Sph2) of BugBuster (EMD Biosciences) containing 20 unit/mL DNase I (Thermo Scientific) and 0.25 mM phenylmethylsulfonyl fluoride (Sigma-Aldrich), and the suspension was swirled for 20 min at room temperature. The lysates were poured into Corex tubes and subjected to centrifugation at 15,000 x g for 20 min at 4°C in a Sorvall RC-5B Superspeed centrifuge.

To wash the Sph1, Sph3, and Sph4 inclusion bodies, the pellets were suspended in 10 mL of a 10-fold dilution of BugBuster. Following centrifugation at 15,000 x g for 20 min at 4°C, the pellets were suspended in 5 mL of 100 mM NaH_2_PO_4_, 10 mM Tris-HCl, and 8M urea, pH 8.0 (Buffer B) and swirled for 15 min at room temperature to solubilize the inclusion bodies. The material was centrifuged at 10,000 x g at room temperature to pellet the cell debris. 1 mL of 50% Ni^2+^-NTA slurry (Qiagen) was added to the solubilized inclusion bodies, and the suspension was mixed for 60 min at room temperature. The material was then poured into a 5-mL polypropylene column (Qiagen) for purification of the recombinant protein. The column was washed with 4 mL Buffer B that was adjusted to pH 6.3 and then with 2 mL Buffer B at pH 5.9. The recombinant protein was eluted with Buffer B adjusted to pH 4.5, and 0.5 mL fractions were collected. The fractions were neutralized by adding 55.5 μl of 1 M Tris-HCl, pH 8.0, to each.

To purify soluble Sph2, 250 μl of 0.5 M imidazole and 2 mL of the 50% Ni^2+^-NTA slurry (Qiagen) were added to 10 mL of lysate. The mixture was mixed for 60 min at 4°C and then poured into a 5 mL polypropylene column. After collecting the flow through, the column was washed at 4°C with a total of 30 mL of 20 mM imidazole in Wash Buffer (50 mM NaH_2_PO_4_, 0.5 M NaCl, pH 8.0). The protein was eluted at 4°C with 0.5 M imidazole in Wash Buffer, and 1 mL fractions were collected. Protein concentrations of all purified Sph proteins were determined using the Pierce BCA Protein Assay kit (Thermo Scientific) with BSA standards.

### RNA extraction and DNase treatment

For preparation of RNA, 25 mL of culture was transferred into an Erlenmeyer flask, quickly chilled by swirling for 7 s in a dry ice-ethanol bath, and centrifuged at 9,200 x g for 20 min at 4°C. RNA was isolated from leptospires as follows: 1 mL Trizol (Life Technologies; Grand Island, New York, USA) was added to the cell pellet, resuspended thoroughly by pipetting followed by incubation at room temperature for 5 min. 200 μL of chloroform (Sigma-Aldrich) was added, mixed vigorously for 15 s followed by incubation at room temperature for 3 min. The tubes were centrifuged at 12,000 x g for 15 min at 4°C, and the upper aqueous layer was pipetted into fresh tubes. RNA was precipitated by the addition of equal volumes of isopropanol, mixed and incubated for 10 min and subjected to centrifugation at 16,000 x g for 15 min at 4°C. The pellet was washed with 1 mL of 75% ethanol, air dried and dissolved in 84 μL of RNase free water. The dissolved RNA was protected from degradation by addition of 1μL of 20 U/μL SUPERase In RNase inhibitor (Life Technologies). DNA was digested by addition of 10 μL of 10X Turbo DNase buffer and 5 μL of Turbo DNase followed by incubation in 37°C water bath for 2 h. The RNA samples were subjected to clean-up using the RNeasy Mini kit (Qiagen; USA) as described in the manufacturer’s instructions. The concentration of the RNA was determined using the NanoVue spectrophotometer (GE Healthcare; Piscataway, New Jersey, USA) and the quality of RNA was assessed by examining the A_260_/A_280_ and A_260_/A_230_ ratios and by electrophoresis of 200 ng of RNA onto a 1.2% agarose gel.

### Quantitative RT-PCR

cDNA was synthesized using the iScript cDNA Synthesis Kit (Bio-Rad) following the manufacturer’s instructions. The reaction was set up in a microcentrifuge tube in a reaction volume of 20 μL containing 4 μL of 5X iScript Reverse Transcription Supermix, 1 μg RNA, and nuclease-free water. cDNA synthesis was carried out in a Master Cycler gradient thermal cycler (Eppendorf; Hauppauge, New York, USA) with priming at 25°C for 5 min followed by reverse transcription at 42°C for 30 min and enzyme inactivation at 85°C for 5 min. A separate reaction without reverse transcriptase was included as a negative control. The synthesized cDNA was diluted with RNase free water (Ambion) to obtain a working concentration of 10 ng/μL.

The quantification of cDNA was done using real-time PCR using iQ5 SYBR Green Supermix (Bio-Rad) and the iQ5 Multicolor Real-time PCR Detection System (Bio-Rad). Each reaction contained cDNA derived from 50 ng of RNA, 10 pmol of each forward and reverse primers, and 12.5 μL of 2X iQ5 SYBR Green supermix in a total volume of 25 μL. The *sph1-*, *sph2*- and *lipL41*-specific primers (Table 1) were designed using Primer Premier (Premier Biosoft; Palo Alto, California, USA). The amplification protocol consisted of an initial denaturation for 15 min (95°C) followed by 40 cycles of amplification (15 s at 95°C, 30 s at 58°C, 30 s at 72°C) and a final extension of 2 min at 72°C. Standard curves were constructed by 5-fold serial dilutions (50 ng to 0.08 ng) of cDNA as template in triplicate. Amplification efficiency was evaluated from the standard curves by determining the E value; results in the range of 90% to 110% were considered to be acceptable. The C_T_ values were normalized to that of *lipL41*, and expression levels were calculated using 2^ΔΔC^_T_ method [42]. The relative fold change was calculated by comparison with the EMJH control. The assay was performed using three biological replicates.

### Hemolytic activity

To assess hemolytic activity qualitatively, 5 μL of culture supernatant fluid was spotted onto BBL Trypticase Soy Agar with 5% sheep red blood cells (TSA II) (Becton Dickinson; Sparks, Maryland, USA). Because magnesium is necessary for sphingomyelinase activity, 100 mM MgCl_2_ was added to the culture supernatant to a final concentration of 10 mM prior to spotting the plates. 0.05 units of *Bacillus cereus* sphingomyelinase (Sigma-Aldrich) was spotted as a positive control. The plates were incubated for 20 hours at 37°C and then at 4°C for at least three days.

The liquid-phase hemolysis assay was set up in a 96 well round-bottomed microtiter plate as reported earlier [20] with some modifications. Briefly, sheep erythrocytes were procured commercially from Quad Five (Ryegate, Montana, USA) as a 50% (v/v) suspension in Alsever's's solution. Erythrocytes were collected by centrifugation at 400 x g for 10 min at 8°C, washed three times with cold PBS (pH 7.4), and resuspended in cold PBS to a final concentration of 10%. Each reaction mixture (200 μL) contained 10 mM MgCl_2_ in PBS, with 40 μL 10% washed sheep erythrocytes and 100 μL of culture supernatant fluid. For background measurements, EMJH with 120 mM sodium chloride replaced the spent medium. Three biological replicates were examined. The hemolysis reaction proceeded at 37°C for 90 min followed by incubation at 4°C for 30 min. The plate was centrifuged at 800 x g in an Eppendorf 5430 centrifuge to pellet intact erythrocytes, and the supernatant fluid from each well was transferred to a flat-bottom 96-well ELISA plate. The plate was read in an iMark Microplate Absorbance Reader (Bio-Rad) at 415 nm. Percent hemolysis was calculated by multiplying the PBS background-subtracted absorbance of the sample by 100 and dividing by the absorbance of the osmotically-lysed erythrocytes.

### Sphingomyelinase assay

Sphingomyelinase activity was measured by a coupled assay using the Amplex Red Sphingomyelinase assay kit (Molecular Probes, Invitrogen, USA) as described in the manufacturer’s instructions. The reactions were set up in 96-well special optics flat clear bottom black polystyrene Microplate (Corning, product # 3720). The reaction mixture (200 μL) contained 100 μL test sample and 100 μL of 100 μM Amplex red reagent (containing 2 U/mL horseradish peroxidase, 0.2 U/mL choline oxidase, 8 U/mL alkaline phosphatase, and 0.5 mM sphingomyelin) (Life Technologies). The reaction proceeded for 90 min at 37°C. The fluorescence was measured at excitation and emission wavelengths of 530 nm and 590 nm respectively using the Synergy2 Multi-Mode Microplate Reader (BioTek; Winooski, Vermont, USA). The background fluorescence was corrected by subtracting the negative control, which contained the reaction buffer without sphingomyelinase. Standard curves were generated with *B*. *cereus* sphingomyelinase, and the measurements were fit by nonlinear regression to a hyperbola (one-site binding) model with GraphPad Prism, version 5.04 (GraphPad Software; La Jolla, California, USA). Experiments were performed with three biological replicates.

### Complementation

For complementation of the *sph2* mutant, the mobilizable *sph2* plasmid pRAT708 was transformed into the diaminopimelic acid auxotroph *E*. *coli* β2163, which expresses the RP4 conjugation machinery [43]. The plasmid was transferred into the *L*. *interrogans sph2* mutant by conjugation as described [44, 45]. Transconjugants were selected on EMJH agar plates containing 40 μg/mL kanamycin and 40 μg/mL spectinomycin. After two weeks of incubation at 30°C, colonies were inoculated into EMJH liquid medium. The insertion site of the *sph2*-containing transposon was identified by nested PCR using primers TnK1 and Deg1 for the first PCR reaction and primers TnkN1 and Tag for the second [45].

### Statistical analysis

All values for hemolytic and sphingomyelinase activities were log transformed prior to statistical analysis to achieve similar variances across all groups. One-way ANOVA was conducted with R version 3.0.3 [46]. The Tukey post test was used for group comparisons.

## Results

### Regulation of *sph2* expression in different strains of *L*. *interrogans*

**Fig 1:**
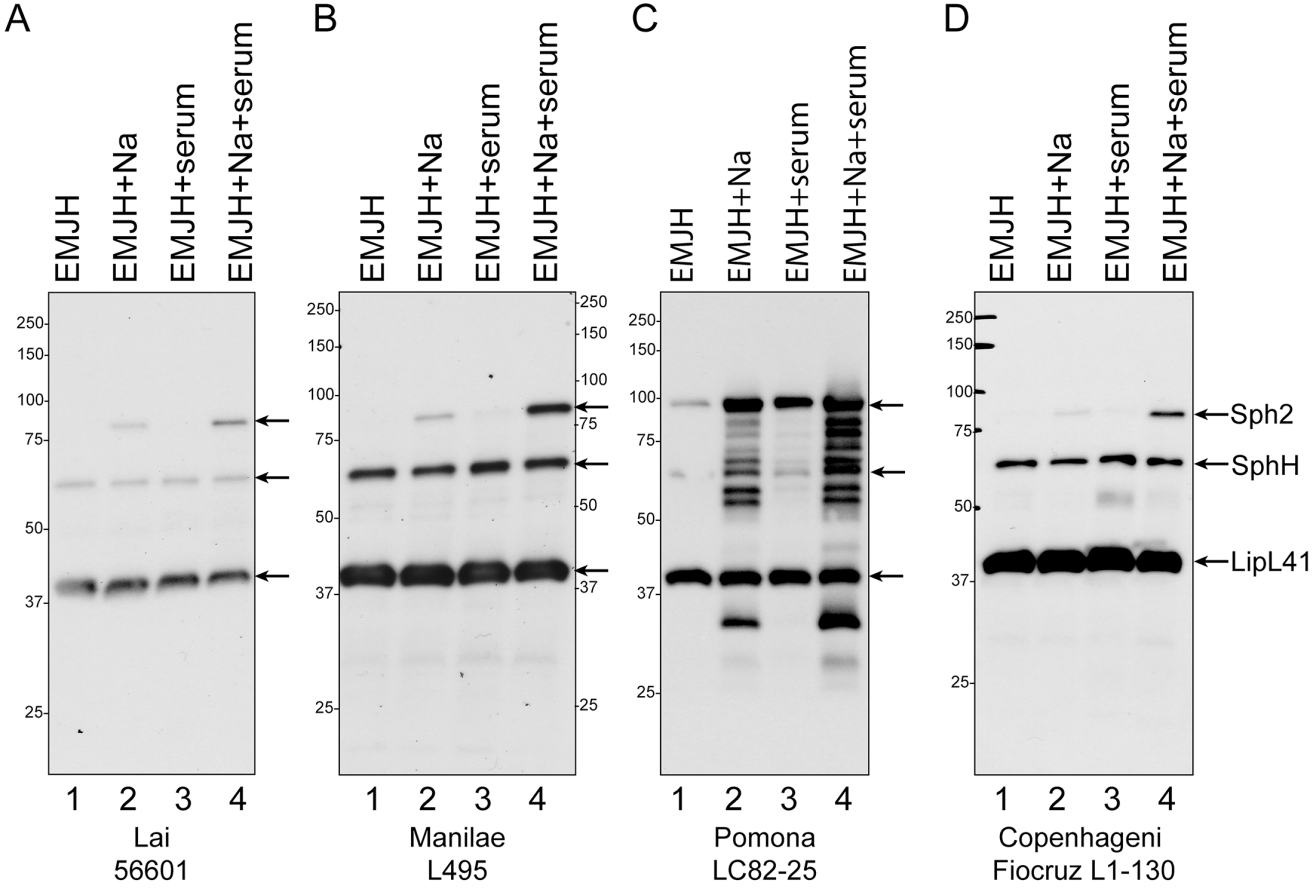
Effect of salt and rat serum on cellular Sph2 production in different strains of *L*. *interrogans*. *L. interrogans* serovar Lai strain 56601 **(A)**, serovar Manilae strain L495 **(B)**, serovar Pomona strain LC82-25 **(C)**, and serovar Copenhageni Fiocruz L1-130 **(D)** were grown in EMJH (lane 1), EMJH supplemented with 120 mM sodium chloride (lane 2) or 10% rat serum (lane 3) or both sodium chloride and rat serum (lane 4) for 4 hours at 30°C. Whole-cell lysates were analyzed by immunoblotting with anti-Sph2 and anti-LipL41 antisera. Arrows identify Sph2, SphH, and LipL41. The anti-Sph2 antiserum cross-reacts with SphH. Positions of molecular mass standards are shown in kilodaltons.

Our earlier work demonstrated that *sph2* gene expression in *L*. *interrogans* strain Fiocruz L1-130 (serovar Copenhageni) increases substantially when incubated in EMJH supplemented with sodium chloride to attain physiological osmolarity [33, 34]. In the current study, we examined *sph2* regulation in three additional strains of *L*. *interrogans*, L495 (serovar Manilae), 56601 (serovar Lai), and LC82-25 (serovar Pomona subtype kennewicki). The Pomona strain was included because members of the serovar produce larger amounts of hemolysin than other serovars [9]. Immunoblots were performed to determine the effects of NaCl and serum on Sph2 protein levels. As shown in Fig 1 (lane 1), Sph2 was not detected in the Fiocruz L1-130 [34], Manilae L495, or Lai 56601 strain when grown in EMJH. The addition of 120 mM sodium chloride to EMJH increased Sph2 production to detectable levels in these three strains (lane 2). On the other hand, Sph2 was detected easily in the Pomona LC82-25 strain growing in EMJH, and Sph2 levels increased further when the medium was supplemented with 120 mM sodium chloride (Fig 1C, lanes 1 and 2). The presence of rat serum to the culture medium had an effect on Sph2 expression independent of osmolarity; Sph2 levels were noticeably higher than that when only sodium chloride was present, despite the osmolarity of the culture medium being similar (Fig 1, lanes 2 and 4). Relative to LipL41 levels, Sph2 levels were substantially higher in the Pomona LC82-25 strain than in the Manilae L495 strain under all conditions (Fig 1). The apparent molecular masses of Sph2 was higher than the calculated masses of 71.0, 71.0, 70.4, and 73.5 kDa in the Copenhageni, Lai, Manilae, and Pomona strains, respectively. The apparent molecular mass of Sph2 in the LC82-25 strain was ~13 kDa higher than those of the other strains (Fig 1). As noted in our earlier study and by others, anti-Sph2 antibodies also recognized SphH in these samples (Fig 1) [32, 34].

**Fig 2:**
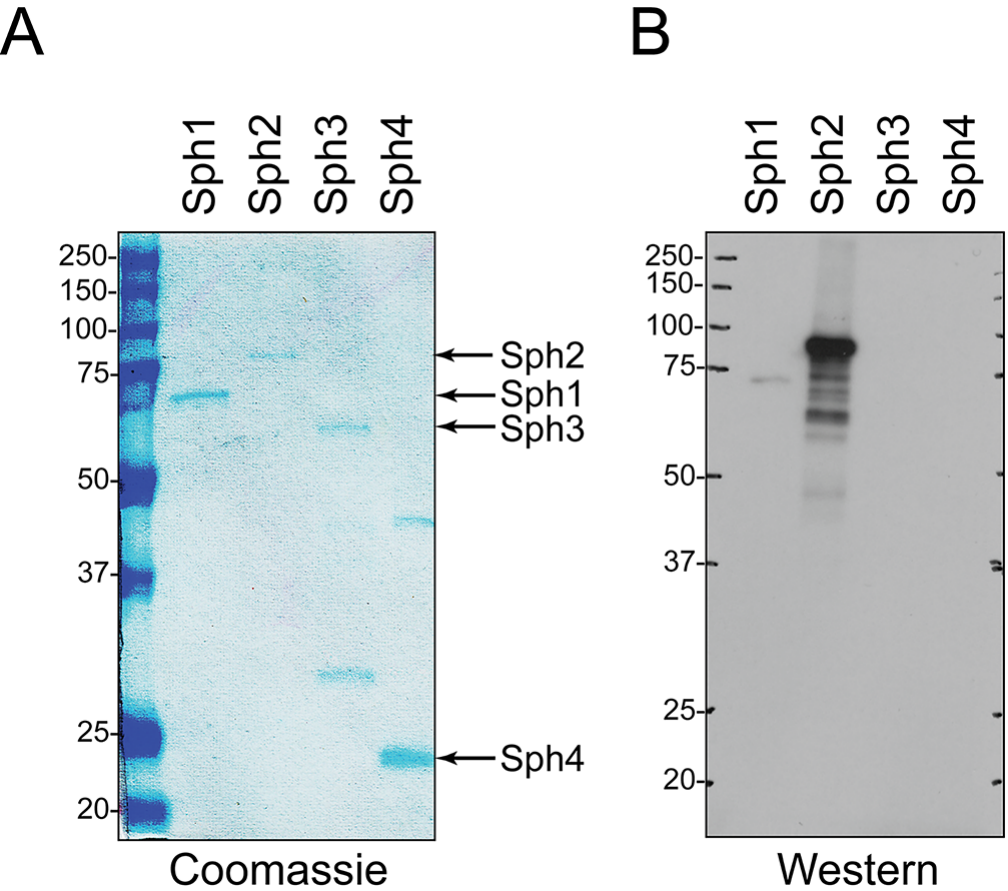
Cross-reaction of anti-Sph2 antibody with other sphingomyelinase-like proteins. 50 ng of recombinant Sph1, Sph2, Sph3, and Sph4 were subjected to SDS-polyacryamide gel electrophoresis in duplicate gels. One gel was stained with Coomassie blue **(A)**, and the second was analyzed by Western blot with anti-Sph2 antiserum **(B)**. Positions of molecular mass standards are shown in kilodaltons.

Sph2 breakdown products were visible by immunoblot after growth of the serovar Pomona strain in 120 mM NaCl with and without serum (Fig 1C). To exclude the possibility that some of the bands originated from the other sphingomyelinase-like proteins, we examined the cross-reactivity of our anti-Sph2 antiserum with purified recombinant forms of Sph1, Sph3, and Sph4. Equal masses of Sph1, Sph2, Sph3, and Sph4 were subjected to SDS-polyacrylamide gel electrophoresis (Fig 2A) and immunoblot analysis with the anti-Sph2 antiserum (Fig 2B). As observed for the native form of Sph2, the recombinant form ran more slowly than expected from its calculated molecular mass, whereas Sph1, Sph3, and Sph4 migrated as expected (Fig 2A). The immunoblot shows that the anti-Sph2 antibody does not cross-react with Sph3 or Sph4 and reacts poorly with Sph1 (Fig 2B). These results indicate that the bands observed below the Sph2 species in the Pomona LC82-25 lysate are Sph2 breakdown products, although we cannot rule out the possibility that the bands running below SphH arose from SphH degradation.

**Fig 3:**
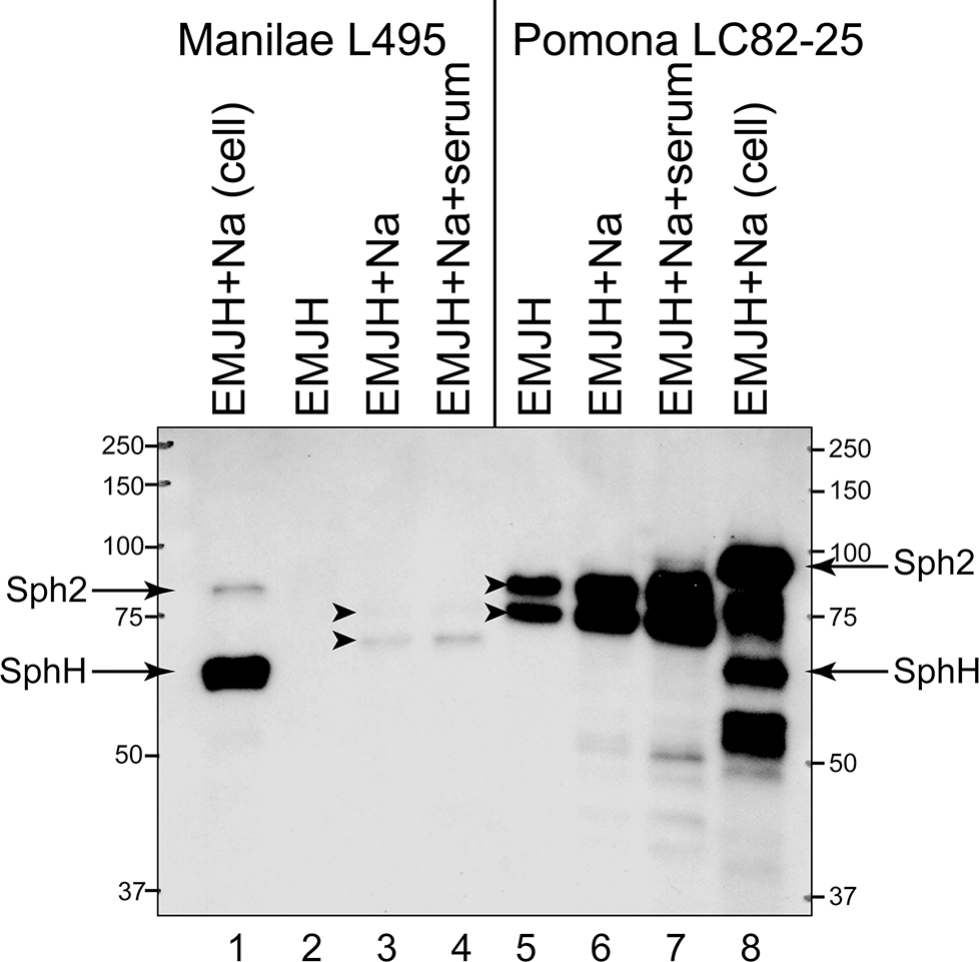
Effect of salt and serum on the amount of Sph2 released by *L*. *interrogans*. Sph2 was recovered by immunoprecipitation from spent growth medium of *L*. *interrogans* Manilae L495 (lanes 2–4) and Pomona LC82-25 (5–7) strains grown under different conditions for 4 hours at 30°C in EMJH (lanes 2 and 5), EMJH with 120 mM sodium chloride (lanes 3 and 6), and EMJH with sodium chloride and 10% rat serum (lanes 4 and 7). Spent medium was collected, and Sph2 was detected by immunoprecipitation (lanes 2–7). Cell lysates are shown for comparison (lanes 1 and 8). The Pomona cell lysate was diluted four fold before loading. Arrows indicate Sph2 and SphH observed in cell lysates. The anti-Sph2 antiserum cross-reacts with SphH. Arrowheads mark Sph2 detected in the spent medium. Positions of molecular mass standards are shown in kilodaltons.

Immunoprecipitation of Sph2 was performed with the spent growth medium from cultures of the Manilae L495 and Pomona LC82-25 strains to assess the effects of salt and serum on the levels of extracellular Sph2. We did not detect Sph2 with the L495 strain grown in EMJH. When EMJH was supplemented with sodium chloride, with or without rat serum, Sph2 released from the L495 strain was detected in two smaller forms (Fig 3, lanes 3 and 4), as shown in our earlier study with Fiocruz L1-130 [30]. The Pomona LC82-25 strain exhibited similar regulation of extracellular Sph2 levels by sodium chloride and rat serum, except the amount of Sph2 detected was much greater than that in the L495 strain (Fig 3, lanes 2–4 vs. 5–7). The two Sph2 bands in the serovar Pomona immunoprecipitates were smaller than the species detected in the corresponding cell lysate (Fig 3, lanes 5–8). The apparent molecular masses of the extracellular forms of Sph2 were greater in the LC82-25 strain than those of the L495 strain, as was the case for the cellular form.

**Fig 4:**
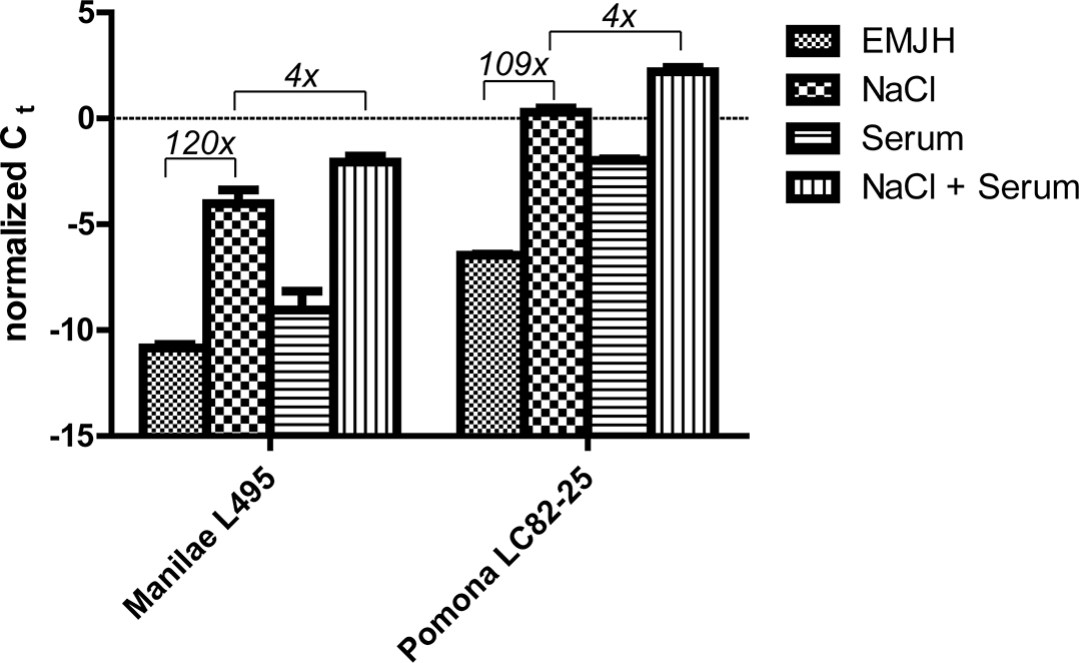
Effect of salt and serum on *sph2* transcript levels in *L*. *interrogans*. Transcript levels were determined by quantitative RT-PCR. C_t_ values for *sph2* transcript were normalized to those for *lipL41* transcript. Fold differences between growth conditions are shown in italics. Mean and standard deviation from three biological replicates are shown; *p* < 0.001 for all group comparisons (one-way ANOVA, Tukey multiple comparisons post-test).

We compared *sph2* transcript levels in the serovar Pomona strain LC82-85 with those of the serovar Manilae strain L495 in response to environmental conditions. Quantitative RT-PCR measurements revealed that when grown in standard EMJH medium *sph2* transcript levels were 21 fold higher in the Pomona strain than in the Manilae strain (Fig 4). In both strains, the addition of 120 mM sodium chloride to the cultures increased *sph2* transcript levels by over 100 fold (Fig 4). At the higher osmolarity, levels of *sph2* transcript remained nearly 20 fold higher in the Pomona strain than in the Manilae strain. Addition of 10% rat serum increased *sph2* transcript levels by four fold. The ~20-fold difference in *sph2* transcript levels between the two strains was maintained when both 10% rat serum and the additional 120 mM sodium chloride were present in the culture medium.

### Regulation of hemolysin and sphingomyelinase production by *L*. *interrogans*

**Fig 5:**
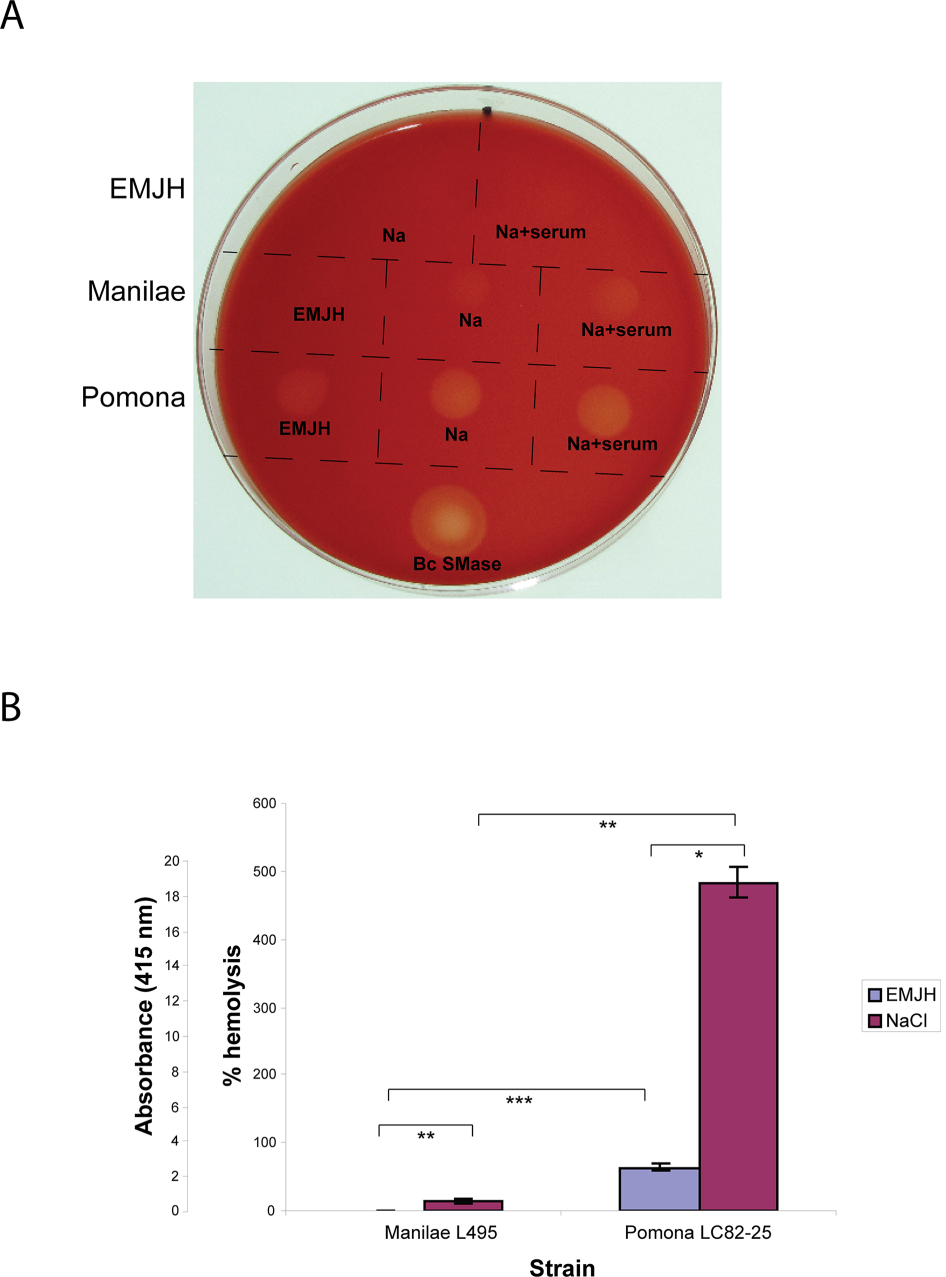
Hemolytic activity in spent growth medium of *L*. *interrogans*. **A.** Hemolytic activity associated with spent growth medium from *L*. *interrogans* Manilae L495 and Pomona LC82-25 strains grown in EMJH, EMJH supplemented with 120 mM sodium chloride (Na) and EMJH supplemented with 120 mM sodium chloride and 10% rat serum (Na+serum) was examined on blood agar plates. 50 mU of *Bacillus cereus* sphingomyelinase (Bc SMase) was spotted onto the agar as a positive control. **B.** Hemolytic activities of spent growth medium from Manilae L495 and Pomona LC82-25 cultures incubated in EMJH and EMJH supplemented with 120 mM sodium chloride were measured by a quantitative hemolytic assay. The spent medium from the Pomona LC82-25 cultures were diluted 10 fold into EMJH plus 120 mM sodium chloride prior to conducting the assay. Measurements obtained with the diluted Pomona samples were multiplied by ten and plotted on the graph. Data are presented as mean ± standard deviation of triplicates. *, *p* < 0.05; **, *p* < 0.01; ***, *p* < 0.001 (one-way ANOVA, Tukey multiple comparisons post-test).

The levels of hemolytic and sphingomyelinase activities in the spent growth medium of the Pomona LC82-25 and Manilae L495 strains grown in EMJH and in EMJH supplemented with 120 mM sodium chloride were determined. To assess hemolytic activity qualitatively, the culture supernatant fluid was spotted onto sheep erythrocyte agar plates. The culture medium, which was adjusted to physiological osmolarity with sodium chloride, did not cause detectable lysis of the erythrocytes (Fig 5A). On the other hand, the spent growth medium from both strains, including those obtained from cultures containing rat serum, caused partial clearance of erythrocytes on the plate (Fig 5A).

**Fig 6:**
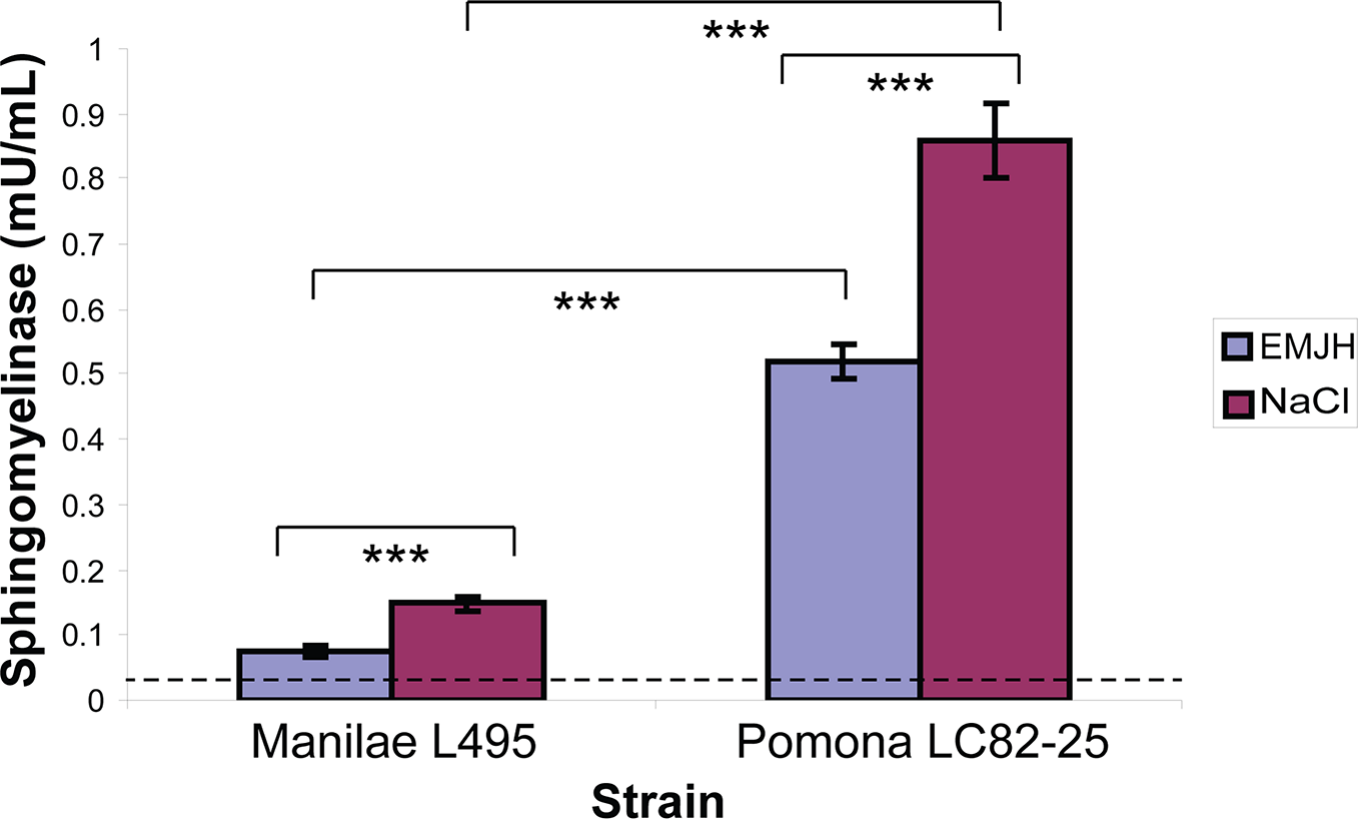
Sphingomyelinase activity in spent growth medium of *L*. *interrogans*. A sphingomyelinase assay was conducted with the spent growth medium of *L*. *interrogans* Manilae L495 and Pomona LC82-25 strains grown in EMJH (blue) and in EMJH supplemented with 120 mM NaCl (purple). The dashed line indicates the background enzymatic activity of EMJH with 120 mM NaCl alone. Data are presented as mean ± standard deviation of three biological replicates; ***, *p* < 0.001 (one-way ANOVA, Tukey multiple comparisons post-test).

The amount of hemolytic activity in the spent growth medium was quantified with a liquid-phase assay. In EMJH, the Pomona strain showed greater hemolytic activity for sheep erythrocytes than the Manilae strain (Fig 5B). Both strains released higher levels of hemolytic activity when EMJH was supplemented with 120 mM sodium chloride. In the process of developing the hemolysis assay, we discovered that hemolytic activity in spent medium from the Pomona strain incubated in EMJH with 120 mM sodium chloride was at least 92% lower when 10% rat serum was also present, even though it was detected in sheep erythrocyte plates (Fig 5A). The hemolysis results with the strains grown in EMJH and EMJH with 120 mM sodium chloride were reflected in an assay for sphingomyelin hydrolase activity (Fig 6). The results from the Western blots, hemolysis assay, and enzymatic assay reveal a correlation between *sph2* expression and extracellular hemolysis and sphingomyelinase activities of *L*. *interrogans*.

### Reduced hemolytic and sphingomyelinase activity secreted by an *L*. *interrogans sph2* mutant

**Fig 7:**
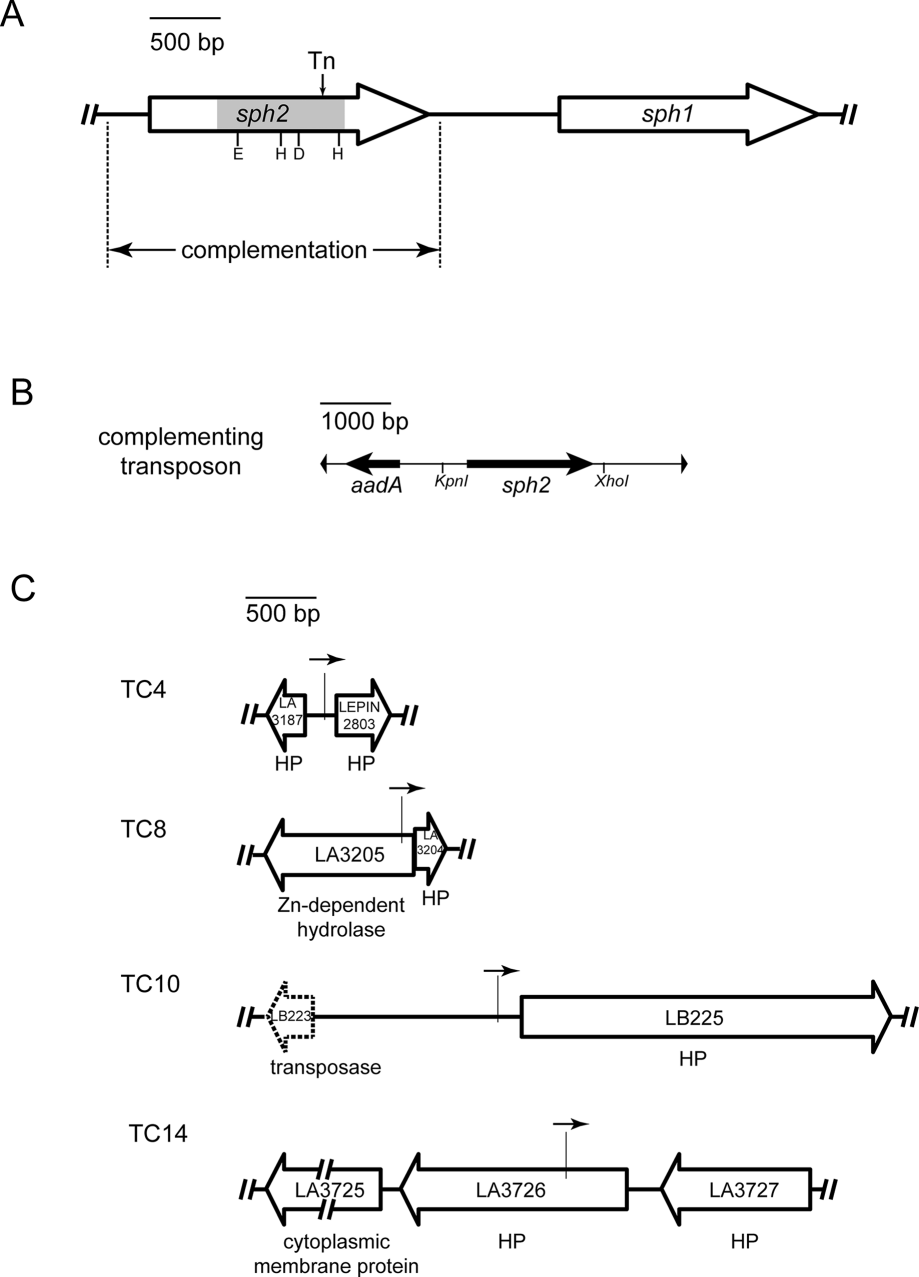
Location of tranposons in an *L*. *interrogans sph2* mutant and in *sph2* complemented isolates. **A.** Genetic organization of *L*. *interrogans sph1*-*sph2* locus. The location of the transposon insertion in the *sph2* mutant is designated with "Tn." The ends of the sequence cloned for complementation studies are marked with dashed lines. The segment of *sph2* encoding the enzymatic domain is shaded gray, and the catalytic residues demonstrated experimentally to be necessary for *Bacillus subtilis* sphingomyelinase activity are abbreviated by their single-letter amino acid codes. **B.** Genetic structure of the complementing transposon. The *sph2* sequences were cloned between the *Kpn*I and *Xho*I restriction sites in the transposon. The *aadA* gene encodes resistance to spectinomycin. **C.** Insertion sites of the complementing transposon. The location of the complementing transposon in four transconjugants (TC) is indicated by the vertical line. The arrow above the vertical line depicts the orientation of the *sph2* gene in the transposon. The dashed open arrow indicates a pseudogene. HP, hypothetical protein.

**Fig 8:**
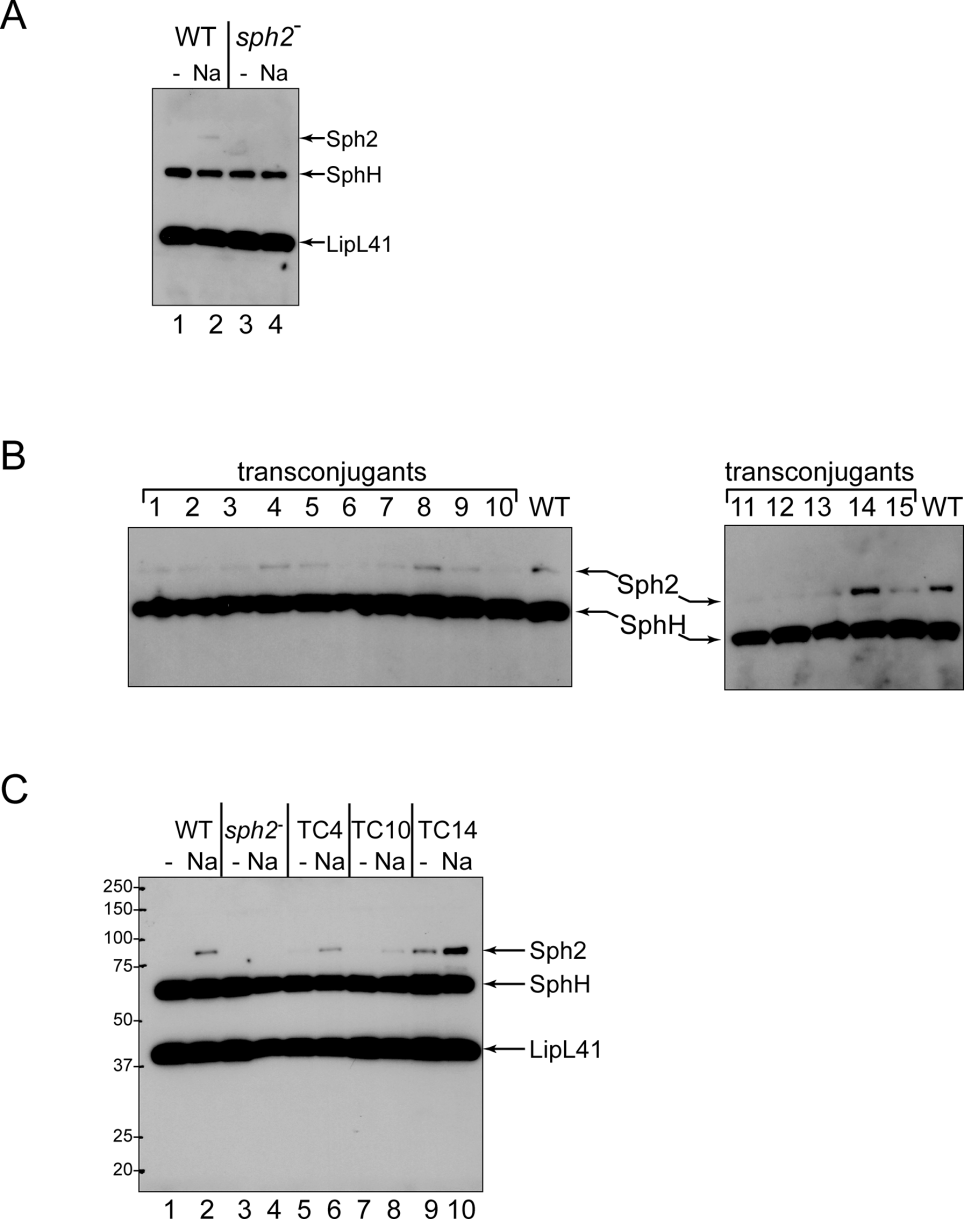
Immunoblot analysis of the *L*. *interrogans sph2* mutant and its complemented isolates. **A.** Immunoblot analysis of the *sph2* mutant. The wild-type (WT) and mutant (*sph2* ^-^) strains were incubated in EMJH (-) or EMJH with 120 mM sodium chloride (Na) for six hours, and cell lysates were examined by immunoblot analysis with anti-Sph2 and anti-LipL41 antisera. The anti-Sph2 antiserum cross-reacts with SphH. **B.** Screen for complemented isolates producing Sph2. The *sph2* gene was introduced into the *sph2* mutant by conjugation. 15 transconjugants were examined for production of Sph2 following incubation in EMJH with 120 mM sodium chloride for six hours. The immunoblot was probed with anti-Sph2 antiserum. **C.** Regulation of Sph2 production in three transconjugants. The transconjugants TC4, TC10, and TC14 were examined for Sph2 production following incubation in EMJH (odd-numbered lanes) and EMJH with 120 mM sodium chloride (even-number lanes). The immunoblot was probed with anti-Sph2 and anti-LipL41 antisera.

To examine the contribution of *sph2* to secreted hemolytic and sphingomyelinase activities by a genetic approach, we were provided with the highly-passaged *L*. *interrogans* strain L391 (serovar Lai) and an isogenic *sph2* mutant, which was generated by transposon insertional mutagenesis [38]. The transposon is located within *sph2* near the end of the segment encoding the enzymatic domain of the protein (Fig 7A). The truncated form predicted to be generated by the mutant allele lacks the second catalytic histidine residue in the enzymatic domain. Substitution of this histidine with alanine in *Bacillus cereus* sphingomyelinase completely abolishes its enzymatic activity [47]. Therefore, the truncated form expressed from the mutant *sph2* allele is unlikely to possess residual enzymatic function. Immunoblot analysis with the *sph2* mutant and its wild-type parent confirmed that the mutant was unable to produce the full-length form of Sph2 following incubation at physiological osmolarity for six hours (Fig 8A, lane 4). The *sph1* coding region lies 915 bp downstream of *sph2* (Fig 7A). Quantitative RT-PCR analysis indicates that *sph1* transcript levels were 34% lower in the *sph2* mutant compared with that in the wild-type strain.

We introduced an intact copy of *sph2* into the mutant for complementation studies. We selected the *sph2* gene from the Fiocruz L1-130 strain because of the possibility that the Sph2 protein synthesized by the highly-passaged L391 strain was not fully active. The Sph2 protein sequences of the Fiocruz L1-130 and Lai 56601 strains are 99.5% identical (621/624), with none of the three differences occurring in predicted catalytic residues [22]. The *sph2* gene was introduced by conjugation on a suicide plasmid carrying *sph2* within a *Himar1* transposon carrying a gene encoding resistance to spectinomycin (Fig 7B). Following mating and incubation on selective plates, 15 transconjugants were screened for Sph2 production. The transconjugants exhibited various levels of Sph2 following incubation at physiologic osmolarity (Fig 8B), as would be expected from random insertion of the complementing transposon. Transconjugants that produced high (TC14), medium (TC4), and low (TC10) levels of Sph2 were further examined for control of Sph2 production (Fig 7C). TC14 showed higher basal levels of Sph2 than the wild-type strain (Fig 8C, lane 9 vs 1), and Sph2 levels increased further upon incubation at physiologic osmolarity (Fig 8C, lane 10). The complementing transposon in TC14 was located in the hypothetical gene LA3726, with *sph2* in the transposon oriented opposite of the disrupted gene (Fig 7C). In TC10, Sph2 levels were regulated by sodium chloride, but the amount produced at physiologic osmolarity was low relative to the levels produced in the wild-type strain (Fig 8C, lane 8 vs 2). The *sph2* gene in the complementing transposon was positioned near the promoter region of the hypothetical gene LB225 with the two genes directed in the same orientation (Fig 7C). TC4 generated similar levels of Sph2 as the wild-type strain (Fig 8C, lanes 5 and 6 vs. 1 and 2). The complementing transposon in TC4 was inserted in the promoter region of the hypothetical gene LEPIN2803 with *sph2* oriented in the same direction (Fig 7C).

**Fig 9:**
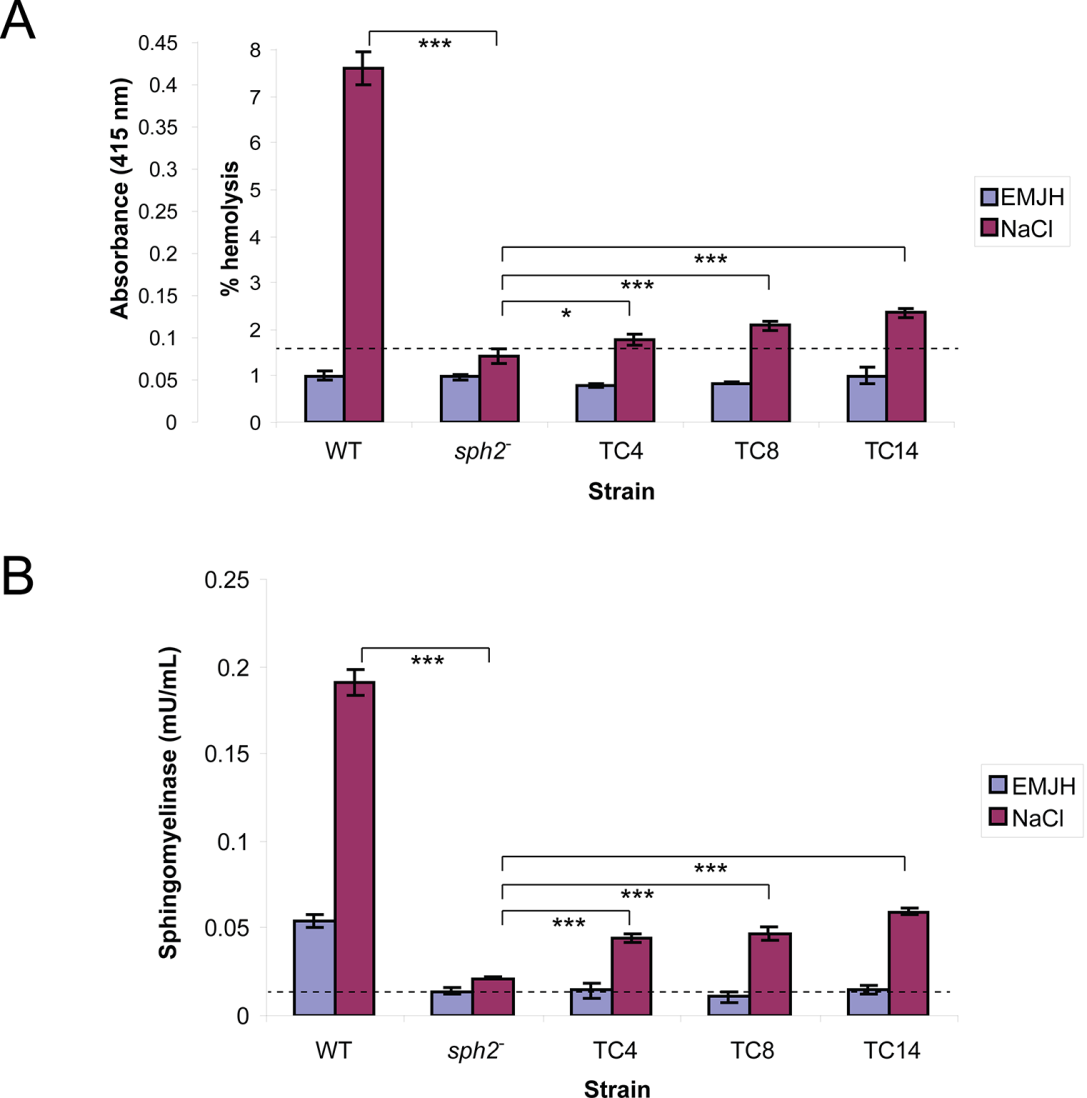
Hemolytic and sphingomyelinase activities secreted from *L*. *interrogans sph2* mutant and complemented isolates. Culture supernatant fluid from wild-type strain Lai L391, *sph2* mutant, and *sph2* mutant complemented with wild-type *sph2* (TC4, TC8, and TC14) incubated in EMJH and EMJH with 120 mM sodium chloride for 24 hours was assayed for hemolytic activity **(A)** and sphingomyelinase activity **(B)**. The dashed line indicates the background activity in culture medium (EMJH plus 120 mM NaCl) alone. Mean and standard deviation from three biological replicates are shown; *, *p* < 0.05; ***, *p* < 0.001 (one-way ANOVA, Tukey multiple comparisons post-test).

For the hemolysis assay, cultures were incubated for 24 hours to permit accumulation of Sph2. Hemolytic activity was higher in spent medium collected from cultures of the wild-type strain incubated at physiological osmolarity compared to incubation in EMJH alone (Fig 9A). However, hemolytic activity remained at background levels in spent medium collected from cultures of the *sph2* mutant (Fig 9A), indicating that *sph2* is required for hemolytic activity. For complementation analysis, three transconjugants were selected for their high levels of Sph2 production at physiologic osmolarity: TC4, TC8, and TC14 (Fig 8B). The complementing transposon in TC8 was inserted in a gene encoding a zinc-dependent hydrolase (LA3205), with *sph2* oriented opposite of the disrupted gene (Fig 7C). The three complemented strains produced significantly more hemolytic activity than the *sph2* mutant (Fig 9A). However, the hemolytic activity secreted by the complemented mutants was less than that generated by the wild-type strain.

The sphingomyelinase activity present in the samples was also quantified. The *sph2* mutation resulted in a substantial reduction of sphingomyelinase activity, although activity above background levels was detected following incubation at physiologic osmolarity (Fig 9B). Similar to what we observed with the hemolysis assay, sphingomyelinase activity was partially restored with the three complemented strains (Fig 9B).

## Discussion

In this study we provide the first experimental evidence that demonstrates that Sph2 contributes to the hemolytic and sphingomyelinase activities secreted by *L*. *interrogans*. The amounts of transcript and protein generated from *sph2* correlated with the levels of hemolysis and sphingomyelinase activities observed in spent culture medium when examining the Manilae L495 and Pomona LC82-25 strains incubated in standard culture medium and in medium supplemented with additional sodium chloride. More importantly, hemolysis associated with the spent growth medium from an *sph2* mutant failed to rise above background levels. Additionally, the *sph2* mutation eliminated most of the sphingomyelinase activity. These results suggest that Sph2 is the dominant hemolysin secreted by *L*. *interrogans* during cultivation in EMJH adjusted to physiologic osmolarity with sodium chloride.

Complementation of the mutant with the *sph2* gene partially restored hemolytic and sphingomyelinase activities in three transconjugants producing similar or higher levels of Sph2 as the wild-type strain. These results confirm that *sph2* contributes to the secreted hemolytic and sphingomyelinase activities, most likely by encoding a single protein that possesses both activities. However, wild-type levels of hemolytic and sphingomyelinase activities were not recovered in the complemented strains. It is unlikely that all three insertions of the complementing transposon disrupted Sph2 secretion or another process necessary for wild-type expression of the two activities. Partial complementation of the hemolytic activity may be explained in part by the mild reduction of *sph1* transcript levels observed in the *sph2* mutant. Another possibility for the incomplete dominance of the wild-type *sph2* gene is that the truncated form of Sph2 produced by the mutant allele interferes with the function of the wild-type protein. We did not detect a truncated Sph2 protein in our immunoblots with the expected molecular mass of 46 kDa. Nevertheless, the same property of Sph2 that causes it to migrate slower than its calculated mass during electrophoresis could also cause the truncated form to run slowly, causing the protein to be obscured by the intense SphH band in the blot.

The *sph2* mutation left behind a trace amount of sphingomyelinase activity above background levels. Our previous sequence analysis of the sphingomyelinase-like paralogs revealed that Sph1 and Sph3 lacked a few of the catalytic residues necessary for sphingomyelin hydrolysis activity [22]. Nevertheless, Sph1 and Sph3 may retain a limited capacity to hydrolyze sphingomyelin that is detected only when the dominant sphingomyelinase, Sph2, is removed genetically or when large amounts of recombinant Sph1 and Sph3 are analyzed [21].

Additional hemolysins may also contribute to hemolytic activity secreted by *L*. *interrogans*, depending on growth conditions. The hemolysins Sph1, Sph2, Sph3, HlpA, and TlyA were detected extracellularly in co-cultures with macrophage-like cell lines in tissue culture medium supplemented with 10% fetal calf serum [16]. Despite the secretion of several hemolysins, our findings predict that Sph2 would be a major hemolysin secreted by *L*. *interrogans* during colonization of the rat host since rat serum increased Sph2 production beyond what was observed without serum at physiologic osmolarity. This finding indicates that undiscovered factors in rat serum are responsible for enhancing *sph2* expression.

As demonstrated in another study [32], we found that a Pomona strain incubated in standard culture medium produced large amounts of Sph2, in contrast to the negligible levels produced by the other four strains tested (Figs 1 and 2 and 6A). For the Pomona and Manilae strains, these results correlated with the secreted hemolytic activity (Fig 4B). The large amount of Sph2 and hemolytic activity observed *in vitro* with a Pomona strain in our study may be implicated in the hemolytic anemia and hemoglobinuria observed in ruminants infected with Pomona strains [12–14]. High basal *sph2* expression may be a consequence of a insertion sequence-like element present near the promoter region of *sph2* or the extra 75 nucleotides located within the 5' end of the coding region in the Pomona strain [18, 22, 48]. The latter sequences, which correspond to one of four tandem 25 amino acid residue repeats lying amino-terminal to the enzymatic domain, are likely to account for the higher molecular masses of the cellular and released forms of Pomona Sph2 relative to that of the other strains, which possess only three repeats. Moreover, the difference in the apparent molecular mass of Sph2 from the Pomona strain versus the other *L*. *interrogans* strains examined in this study is much greater than the calculated difference of 3 kDa. This observation indicates that the unexpectedly slow migration of Sph2 in acrylamide gels observed in this and in our earlier study is related to the repeats, which cause an increase of the apparent molecular mass of Sph2 relative to its calculated mass of up to 18 kDa [34]. The repeats are rich in proline, which could place conformational constraints on the protein to retard its migration through the acrylamide matrix.

Hemolytic activity was detected easily when spent medium from cultures containing rat serum was spotted onto agar plates containing sheep erythrocytes. However, despite the large amount of Sph2 released by the Pomona strain, hemolytic activity measured with the liquid phase assay was reduced considerably when rat serum was present. The inhibitory effect of rabbit and bovine serum on leptospiral hemolytic activity has been noted by others [8, 32, 49]. The ability to detect hemolytic activity in the presence of rat serum on erythrocyte agar plates suggests that the inhibitor is labile or highly diffusible in agar. The hemolysin inhibitor in bovine serum was shown to be phosphatidylethanolamine, phosphotidylcholine, and sphingomyelin [50]. In addition, because rats are a reservoir host for *Leptospira*, rat serum may contain anti-Sph2 antibodies that inhibit the function of Sph2. Our results raise the question of whether Sph2 can function as a hemolysin during hematogenous dissemination. It is possible that uncharacterized host or bacterial processes minimize the inhibitory action of phospholipids during leptospiral infection. Alternatively, the critical function of Sph2 during infection may not involve hemolysis. For example, Sph2 may function as an adhesin or trigger host signaling pathways via its sphingomyelinase when expressed by leptospires that colonize the renal tubule, where plasma proteins that carry phospholipids are likely to be excluded by the glomerulus [22, 31].

Several studies have demonstrated that most of the hemolytic activity detected in *Leptospira* cultures is present in the culture supernatant fluid [8, 9, 11, 32]. Similarly, almost all of the sphingomyelinase activity in another strain of Pomona is extracellular [51]. Although we did not measure the hemolytic and sphingomyelinase activities that remained associated with *L*. *interrogans* cells, the earlier studies suggest that the full-length Sph2 protein that is associated with the bacterial cell remains inactive until secretion, despite the full-length recombinant protein being active *in vitro* [16, 20]. These observations suggest that Sph2 and other hemolysins such as the pore-former SphH [17] are inaccessible to the surface, tied up in the secretion apparatus, or bound by an inhibitor until released from the cell, perhaps to prevent injury to the bacterium. Our findings presented in Fig 3 suggest that processing of Sph2 occurs during its release from the cell and that at least one of the two truncated forms of Sph2 detected extracellularly in *L*. *interrogans* cultures are functional rather than degradation products. However, we cannot rule out the possibility that the true active secreted product is the full-length species that degrades to inactive truncated forms during the lengthy immunoprecipitation procedure. Definitive proof will require demonstration of hemolytic and sphingomyelinase activities of purified forms of the truncated Sph2 species.

The pathway leading to secretion and processing of Sph2 remains to be identified. The processing sites leading to the released forms of Sph2 are predicted to lie within its N- or C-terminal domains, outside of the enzymatic domain. Sph2 appears to lack a classic amino-terminal signal peptide. Given the presence of genes encoding TolC, HlyB, and HlyD orthologs in the *L*. *interrogans* genome [1], secretion of Sph2 and the other sphingomyelinase-like proteins may involve a type I secretion pathway. In one study, a TolC homolog was co-immunoprecipitated using anti-Sph3 immunoglobulin, providing some experimental support for a TolC-based secretion system for the leptospiral sphingomyelinase-like proteins [29].

In conclusion, we report that *sph2* expression and Sph2 secretion are regulated by salt and rat serum. Salt also regulated the levels of hemolytic and sphingomyelinase activities, potentially pathogenic functions of *L*. *interrogans*. These findings indicate that the quantities of hemolytic and sphingomyelinase activities measured in standard cultures of *L*. *interrogans* may underestimate the levels produced during infection, even for strains that produce large amounts in culture. Partial restoration of hemolytic and sphingomyelinase activities of an *sph2* mutant by introduction of an intact *sph2* gene strongly suggests that Sph2 is secreted as an active hemolysin and sphingomyelinase. Given the profound response of *sph2* to host tissue-like conditions and the potential importance of Sph2 in leptospiral pathogenesis, further studies are needed to better understand the molecular mechanisms of *sph2* gene regulation and secretion of its product.
